# Supplemental magnolol or honokiol attenuates adverse effects in broilers infected with *Salmonella pullorum* by modulating mucosal gene expression and the gut microbiota

**DOI:** 10.1186/s40104-021-00611-0

**Published:** 2021-08-09

**Authors:** Fang Chen, Hao Zhang, Encun Du, Qiwen Fan, Na Zhao, Feng Jin, Wei Zhang, Wanzheng Guo, Shaowen Huang, Jintao Wei

**Affiliations:** 1 Institute of Animal Husbandry and Veterinary, Hubei Academy of Agricultural Sciences, Wuhan, China; 2 Hubei Key Laboratory of Animal Embryo and Molecular Breeding, Wuhan, China; 3 Key Laboratory of Prevention and Control Agents for Animal Bacteriosis (Ministry of Agriculture and Rural Affairs), Wuhan, China

**Keywords:** Broiler, Gut microbiota, Honokiol, Immune, Magnolol, *Salmonella pullorum*

## Abstract

**Background:**

*Salmonella pullorum* is one of the most harmful pathogens to avian species. Magnolol and honokiol, natural compounds extracted from *Magnolia officinalis*, exerts anti-inflammatory, anti-oxidant and antibacterial activities. This study was conducted to evaluate the effects of dietary supplemental magnolol and honokiol in broilers infected with *S. pullorum*. A total of 360 one-day-old broilers were selected and randomly divided into four groups with six replicates: the negative control group (CTL), *S. pullorum*-infected group (SP), and the *S. pullorum*-infected group supplemented with 300 mg/kg honokiol (SPH) or magnolol (SPM).

**Results:**

The results showed that challenging with *S. pullorum* impaired growth performance in broilers, as indicated by the observed decreases in body weight (*P* < 0.05) and average daily gains (*P* < 0.05), along with increased spleen (*P* < 0.01) and bursa of Fabricus weights (*P* < 0.05), serum globulin contents, and the decreased intestine villus height and villus/crypt ratios (*P* < 0.05). Notably, supplemental magnolol and honokiol attenuated these adverse changes, and the effects of magnolol were better than those of honokiol. Therefore, we performed RNA-Seq in ileum tissues and 16S rRNA gene sequencing of ileum bacteria. Our analysis revealed that magnolol increased the α-diversity (observed species, Chao1, ACE, and PD whole tree) and β-diversity of the ileum bacteria (*P* < 0.05). In addition, magnolol supplementation increased the abundance of *Lactobacillus* (*P* < 0.01) and decreased unidentified *Cyanobacteria* (*P* < 0.05) both at d 14 and d 21. Further study confirmed that differentially expressed genes induced by magnolol and honokiol supplementation enriched in cytokine-cytokine receptor interactions, in the intestinal immune network for IgA production, and in the cell adhesion molecule pathways.

**Conclusions:**

Supplemental magnolol and honokiol alleviated *S. pullorum-*induced impairments in growth performance, and the effect of magnolol was better than that of honokiol, which could be partially due to magnolol’s ability to improve the intestinal microbial and mucosal barrier.

**Supplementary Information:**

The online version contains supplementary material available at 10.1186/s40104-021-00611-0.

## Background

*Salmonella pullorum* is one of the most harmful pathogens in poultry, leading to high economic losses in developing countries because of its extensive transmission routes [1]. *S. pullorum* can cause intestinal injury, acute systemic disease, compromised production performance, and high mortality in young chickens [2]. In the past several decades, antibiotics have been used for the prevention and treatment of pathogenic infections. However, with the widespread use of antibiotics in livestock production, antibiotic resistance is a growing threat to food safety and public health [3]. Therefore, safe and effective alternative treatment strategies are necessary.

In recent years, plant extracts have been used as potential approaches to protect poultry against bacterial challenges [4, 5]. Magnolol and its isomer honokiol are the main phenolic substances extracted from the root and bark of *Magnolia officinalis*. Magnolol and honokiol are involved in anti-inflammatory, antioxidant, and antibacterial physiological activities [6], and metabolic regulation [7]. In addition, many studies have shown that magnolol and honokiol are highly safe for oral intake [8]. A previous study showed that magnolol could increase the growth performance and improve the meat quality of ducks [9]. Therefore, magnolol and honokiol may be an alternative strategy for the prevention and treatment of pathogenic bacterial infections. Although magnolol and honokiol are isomers of each other, remarkably, there are certain differences in some of their biological functions [10]. However, there is little information about the effects of dietary supplementation with magnolol and honokiol in chicks and whether their effects are different.

The avian intestinal tract provides a biological environment for nutrient digestion and absorption, and plays a crucial role in protecting against pathogens and toxins. Additionally, the intestinal microbiota plays a pivotal role in nutrient delivery and the maintenance of multiple physiological processes related to host health. However, it is not clear whether magnolol and honokiol supplementation can moderate the gut microbial and mucosal barriers. Hence, the present study was performed to evaluate the effects of dietary magnolol and honokiol supplementation on the growth performance and intestinal health of chicks challenged with *S. pullorum.*

## Methods

### Experimental animals and diet

A total of 360 one-day-old Arbor Acres (AA) broilers were randomly assigned to four dietary treatments, with six replicates of 15 chicks. The four treatments were designed as follows: (1) the negative control group with a basal diet, free of any challenges (CTL); (2) the *S. pullorum-*challenged control group (SP); (3) chicks that received a basal diet supplemented with 300 mg/kg honokiol and treated with *S. pullorum* (SPH); and (4) chicks that received a diet supplemented with 300 mg/kg magnolol and treated with *S. pullorum* (SPM). The basal diet was a standard maize/soybean meal diet (Additional file 1). The chicks of each replicate were housed in wire cages (100 cm × 70 cm × 60 cm) in an environmentally controlled house. The CTL group and *S. pullorum* challenge group cages were kept at a certain distance and reared separately to prevent infection. The trial lasted for 21 d.

### Oral challenge and performance

The frozen *S. pullorum* stain (C79–3) was thawed and cultured in Luria-Bertani (LB) broth to activate for three times (37 °C, 16 h). After activating bacteria, expanding propagating and centrifugation, *S. pullorum* was resuspended in sterilized PBS and counted by plate cultivation. Chicks in the SP, SPH, and SPM groups were orally treated with a 0.5 mL (4 × 10^8^ CFU/mL) *S. pullorum* solution at 5 days of age, while chicks in the CTL group received the same amount of sterilized PBS at the same age.

### Sample collection

The supplied and residual feed intakes of each replicate were recorded weekly to calculate the feed conversion ratio (FCR) and average daily feed intake (ADFI). Body weight (BW) and average daily gain (ADG) were measured on days 14 and d 21. At 14 and 21 days of age, one chick from each replicate was randomly selected to be weighed and slaughtered by jugular exsanguination after a 12-h fasting period. The weights of the liver, spleen, and bursa of Fabricus were recorded. Blood samples were collected and centrifuged to separate the serum samples. A 1-cm long section from the distal parts of the jejunum and ileum was collected and fixed in a 10% neutral buffered formalin solution for the histological studies. The tissue and content samples of the ileum were collected and frozen in liquid nitrogen until their use.

### Analyses of serum biochemical indices

Total protein (TP), albumin, globulin, aspartate aminotransferase (AST), alanine aminotransferase (ALT), alkaline phosphatase (ALP), and triglyceride were determined using a colorimetric method (UV-2550, Shimadzu, Japan) with the aid of a commercial kit (Nanjing Jiancheng Institute of Bioengineering, Jiangsu, China).

### Histological studies

After being fixed in the formalin solution for 24 h, the intestinal tissues were embedded in paraffin and sectioned. The sections were then stained with hematoxylin and eosin (HE). Eight complete intestinal villi of each slice were randomly selected to measure the villus height, crypt depth, and thickness of the intestinal muscularis using a micro-image processing system (Shineso, Hangzhou, China).

### 16S rDNA gene sequencing of the ileum microbiome

The microbial genomic DNA extraction from the cecal content samples was carried out using the hexadecyltrimethylammonium bromide (CTAB) method. Using genomic DNA with the required purity and concentration as templates, the V3 and V4 hypervariable regions of the microbial 16S rDNA gene were amplified using primers 341F (5′-CCTACGGGRBGCASCAG-3′) and 806R (5′- GGACTACNNGGGTATCTAAT-3′). The indexed adapters were added to the ends of the 16S rDNA amplicons to generate indexed libraries ready for sequencing on an Illumina NovaSeq 6000 platform (Illumina, San Diego, USA) performed by Novogene Co., Ltd. (Beijing, China). The obtained clean sequences were aligned into operational taxonomic units (OTUs) with a 97% similarity. A species annotation analysis was carried out against the OUTs using the SSUrRNA database of Silva 123 (http://www.arb-silva.de/) and threshold value set as 0.8 ~ 1. Alpha diversity, including observed species, Shannon, Simpson, Chao1, abundance coverage-based estimator (ACE) and PD whole tree, and beta diversity were analyzed using QIIME (Version 1.9.1) [11]. Principal Co-ordinates Analysis (PCoA) plots were generated according to the unweighted Unifrac distance metrics. Differences between groups were analyzed using the Wilcoxon test. Microbial predictive functional profiles generated from 16S rRNA marker gene sequences using Tax4Fun [12] based on KEGG ortholog prediction according to the KEGG pathway database and analyzed using the t-test.

### RNA-Seq analysis of the ileum

Total RNA was isolated from the ileum using the TRIzol reagent (Invitrogen, United States). The purity of the total RNA was analyzed using a NanoPhotometer spectrophotometer (IMPLEN, CA, USA). RNA integrity was assessed using the Bioanalyzer 2100 system (Agilent, CA, USA). The cDNA library was constructed using the NEBNext UltraTM RNA Library Prep Kit for Illumina. The library preparations were sequenced on an Illumina NovaSeq 6000 platform (Illumina, San Diego, USA) performed by Novogene Co., Ltd. (Beijing, China). Clean reads were obtained by removing reads containing adapters and mapping them to the reference genome. Differential expression analysis was performed using the DESeq2 R package (1.16.1). Genes with an adjusted *P*-value < 0.05, and log_2_ (fold-change) > 0.5, or log_2_ (fold-change) < − 0.5 as determined by DESeq2 were considered differentially expressed. A KEGG enrichment analysis of differentially expressed genes was performed using the clusterProfiler R package (3.4.4).

To confirm the RNA-Seq results, we selected seven differentially expressed genes (DEGs) including spermidine-spermine acetyltransferase 1 (*SAT1*, accession: NM_204186), alkaline sphingomyelinase (*ENPP7*, accession: XM_015295596), chemokine ligand 19 (*CCL19*, accession: NM_001302168), chemokine receptor 7 (*CCR7*, accession: NM_001198752), joining chain of multimeric IgA and IgM (*JCHAIN*, accession: NM_204263), claudin 1(*CLDN1*, accession: NM_001013611.2), claudin 5 (*CLDN5* accession: NM_204201.1) for confirmation using qRT-PCR, which was performed using TB Green Premix Ex Taq II (Takara, Dalian, China) by LightCycler 96 PCR (Roche, Mannheim, Germany). The primers used are shown in Additional file 2. The relative quantification of the gene expression was determined using 2^-∆∆Ct^ normalizing to β-actin.

### Statistical analysis

Experimental data on growth performance, immune organ index, serum biochemical index, intestinal histomorphology, and the relative quantification of genes were detected using one-way ANOVA followed by Duncan’s multiple comparison tests using SPSS software (version 20.0; IBM Inc., NY, USA). Differences were considered significantly at *P* < 0.05 while 0.05 < *P* < 0.10 was considered a trend towards significance.

## Results

### Growth performance

The effects of magnolol and honokiol on the growth performance of broilers challenged with *S. pullorum* are shown in Table 1. *S. pullorum* infection significantly decreased the broilers’ average weight and ADG at 14 d (*P* < 0.01), and tended to decrease at 21 d (*P* < 0.1). Magnolol supplementation increased their average weight and ADG at 14 d (*P* < 0.01). In addition, the SP group had a higher FCR on d 1 to 21 compared to the other three groups.

**Table 1:** Effects of magnolol and honokil on growth performance of broilers infected with *S. pullorum*

	CTL	SP	SPH	SPM	SEM	*P*-value
BW (14 d), g	379.80^a^	343.07^c^	357.04^bc^	366.98^ab^	4.33	< 0.01
FCR (1–14 d)	1.51	1.56	1.51	1.49	0.02	0.79
ADG (1–14 d), g	23.80^a^	21.17^c^	22.17^bc^	22.88^ab^	0.31	< 0.01
ADFI (1–14 d), g	36.05	33.43	33.32	34.16	0.49	0.15
BW (21 d), g	694.74^a^	636.84^b^	676.18^ab^	678.56^ab^	7.80	0.07
FCR (1–21 d)	1.51^a^	1.67^b^	1.53^a^	1.51^a^	0.02	0.02
ADG (1–21 d), g	31.08^a^	28.33^b^	30.20^ab^	30.31^ab^	0.37	0.07
ADFI (1–21 d), g	46.84	47.34	46.25	45.91	0.62	0.89

*BW* body weight, *FCR* feed conversion, *ADG* average daily gain, *ADFI* average daily feed intake. All values are expressed as the means (*n* = 6). Means not sharing a common superscript letter within the same row differ significantly (*P* < 0.05)

### Immune organ index

The effects of magnolol and honokiol on the immune organ index of the broilers that were challenged with *S. pullorum* are shown in Table 2. At d 14, *S. pullorum* infection and the supplementation of honokiol and magnolol had no effects on liver weight or liver index (*P* > 0.1). The SP group showed an increased spleen weight, spleen index (*P* < 0.01), and bursa of Fabricius weight (*P* < 0.05) compared to the other three groups. The bursa of Fabricius index in the CTL and SPH groups was lower than that in the SP group (*P* < 0.05). At d 21, the above indices were not significantly different among the four groups (*P* > 0.1).

**Table 2:** Effects of magnolol and honokil on immune organ index of broilers infected with *S. pullorum*

	CTL	SP	SPH	SPM	SEM	*P*-value
d 14						
Liver weight, g	13.14	13.09	12.37	12.78	0.20	0.52
Liver index	3.44	3.57	3.49	3.61	0.05	0.71
Spleen weight, g	0.32^b^	0.47^a^	0.30^b^	0.36^b^	0.02	< 0.01
Spleen index	0.08^b^	0.13^a^	0.08^b^	0.10^b^	0.005	< 0.01
Bursa of Fabricus weight, g	0.77^b^	0.99^a^	0.77^b^	0.83^b^	0.03	0.03
Bursa of Fabricus index	0.20^b^	0.27^a^	0.22^b^	0.23^ab^	0.01	0.02
d 21						
Liver weight, g	25.06	25.77	23.15	22.77	0.69	0.40
Liver index	3.66	3.79	3.48	3.30	0.10	0.34
Spleen weight, g	0.52	0.83	0.77	0.73	0.06	0.23
Spleen index	0.08	0.12	0.12	0.11	0.01	0.27
Bursa of Fabricus weight, g	1.70	1.94	1.70	1.54	0.07	0.35
Bursa of Fabricus index	0.25	0.29	0.25	0.22	0.01	0.34

All values are expressed as the means (*n* = 6). Means not sharing a common superscript letter within the same row differ significantly (*P* < 0.05)

### Serum biochemical index

The effects of magnolol and honokiol on the serum biochemical indices of the broilers challenged with *S. pullorum* are shown in Table 3. *S. pullorum* infection and the supplementation of honokiol and magnolol had no effects on the levels of albumin / globulin (A/G), AST, ALT, and triglyceride at the two time points (*P* > 0.1). Compared to the SP group, the CTL and SPM group had lower levels of TP and globulin (*P* < 0.05) at d 14. The SPH group tended to have an increased level of albumin at d 21 (*P* = 0.08).

**Table 3:** Effects of magnolol and honokil on serum biochemical index of broilers infected with *S. pullorum*

	C	SP	SPH	SPM	SEM	*P-*values
d 14						
TP, g/L	2.17^b^	2.54^a^	2.43^ab^	2.20^b^	0.05	0.03
Albumin, g/L	1.31	1.45	1.37	1.30	0.03	0.30
Globulin, g/L	0.86^b^	1.09^a^	1.06^a^	0.90^b^	0.03	0.02
A/G	1.52	1.33	1.31	1.45	0.04	0.22
AST, U/L	52.20	56.40	55.00	46.33	3.84	0.81
ALT, U/L	4.00	4.17	4.83	3.83	0.30	0.69
ALP, U/L	8,941.00	7,208.20	6,935.00	9,709.67	640.06	0.40
Triglyceride, mg/L	86.59	95.81	93.19	66.82	6.68	0.43
d 21						
TP, g/L	2.40	2.37	2.51	2.31	0.04	0.33
Albumin, g/L	1.36	1.37	1.48	1.36	0.2	0.08
Globulin, g/L	1.04	1.01	1.03	0.95	0.03	0.67
A/G	1.34	1.36	1.45	1.43	0.03	0.51
AST, U/L	31.00	56.83	63.83	58.75	6.82	0.31
ALT, U/L	3.33	4.50	5.00	3.80	0.34	0.33
ALP, U/L	9,229.40	7,340.75	9,136.40	8,072.40	602.77	0.70
Triglyceride, mg/L	57.98	52.62	54.01	54.87	3.49	0.96

*TP* total protein, *A/G* albumin/ globulin, *AST* aspartate aminotransferase, *ALT* alanine aminotransferase, *ALP* alkaline phosphatase; All values are expressed as the means (*n* = 6). Means not sharing a common superscript letter within the same row differ significantly (*P* < 0.05)

### Intestinal histomorphology

The effects of magnolol and honokiol on the intestinal histomorphology of the broilers challenged with *S. pullorum* are shown in Table 4. At d 14, the villus height of the SP group was lower than that of the SPH and SPM groups (*P* < 0.05), while the SP group showed the lowest V/C in the jejunum and ileum (*P* < 0.01). At day 21, the jejunum villus height of the SP group was lower than that of the CTL and SPM groups (*P* < 0.05), while the ileum villus height of the SPM group was higher than that in the SP and SPH groups (*P* < 0.05). In addition, the SPM group had the highest V/C values compared to the other groups (*P* < 0.01). *S. pullorum* infection and the supplementation of honokiol and magnolol had no effect on the crypt depth and the thickness of the intestinal wall at d 14 and d 21 (*P* > 0.1).

**Table 4:** Effects of magnolol and honokil on intestinal histomorphology of broilers infected with *S. pullorum*

	C	SP	SPH	SPM	SEM	*P-*values
d 14						
Jejunum						
Villus height, μm	1,226.63^ab^	1,031.56^b^	1,295.84^a^	1,255.41^a^	36.52	0.03
Crypt depth, μm	220.89	250.00	202.88	202.26	8.32	0.34
intestinal wall thickness, μm	373.46	347.48	374.85	329.44	17.12	0.72
Villus/Crypt	5.78^a^	4.24^b^	6.60^a^	6.34^a^	0.26	< 0.01
Ileum						
Villus height, μm	669.53^ab^	573.94^b^	728.16^a^	744.77^a^	22.06	0.02
Crypt depth, μm	153.39	152.81	124.64	145.03	5.79	0.36
intestinal wall thickness, μm	270.83	238.03	200.1	244.3	15.23	0.51
Villus/Crypt	4.58^b^	3.92^bc^	6.00^a^	5.28^ab^	0.20	< 0.01
d 21						
Jejunum						
Villus height, μm	1,219.2^a^	994^b^	1,143.29^ab^	1,291.89^a^	41.14	0.05
Crypt depth, μm	249.85	201.54	224.2	223.24	9.93	0.38
intestinal wall thickness, μm	347.85	278.86	263.02	314.14	14.38	0.17
Villus/Crypt	4.99	4.99	5.45	6.32	0.34	0.42
Ileum						
Villus height, μm	770.11^ab^	643.84^b^	736.32^b^	900.72^a^	32.25	0.03
Crypt depth, μm	153.75	143.64	155.27	149.78	6.28	0.94
intestinal wall thickness, μm	280.56	248.89	265.04	247.79	11.58	0.75
Villus/Crypt	5.18^b^	4.66^b^	4.92^b^	6.11^a^	0.15	< 0.01

All values are expressed as the means (*n* = 6). Means not sharing a common superscript letter within the same row differ significantly (*P* < 0.05)

### Microbial composition of the ileum

**Fig. 1:**
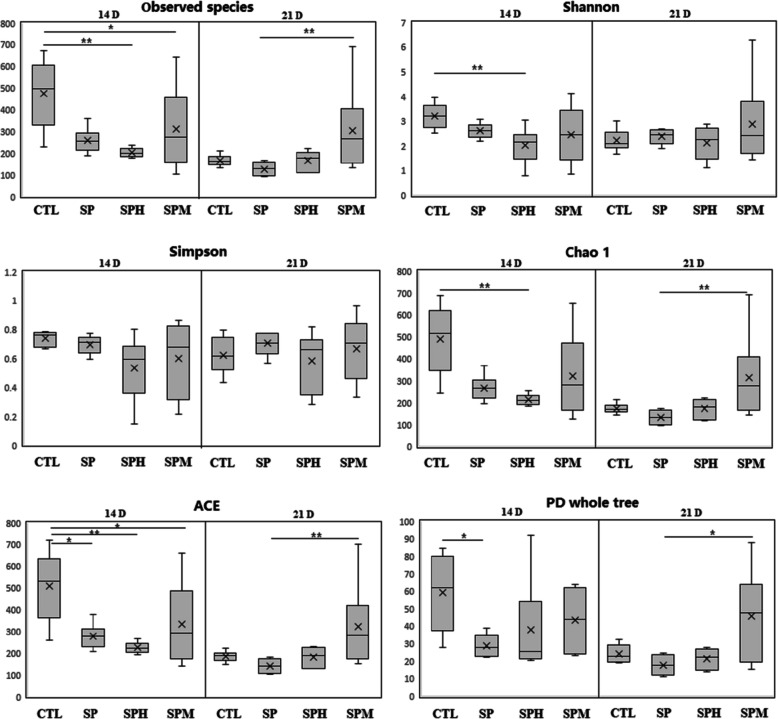
Alpha diversity of the ileum microbiota of briolers. * means *P* < 0.05; ** means *P* < 0.01. Box plots show means and quartiles (*n* = 6)

The α-diversity, including observed species, Shannon, Simpson, Chao1, ACE, and PD whole tree index of ileal microbiota is shown in Fig. 1. At d 14, the observed species, Shannon, and Chao1 estimators of the ileal microbiota in the chicks from the SPH group were lower than those in the CTL group. Compared to the CTL group, the PD whole tree index of microbiota decreased in the SP group (*P* < 0.05). Decreased ACE occurred in all three *S. pullorum-*infected groups. At d 21, SPM group had high level of the number of observed species, and the indices of Chao1, ACE, and PD whole tree compared to the SP group (*P* < 0.05).

**Fig. 2:**
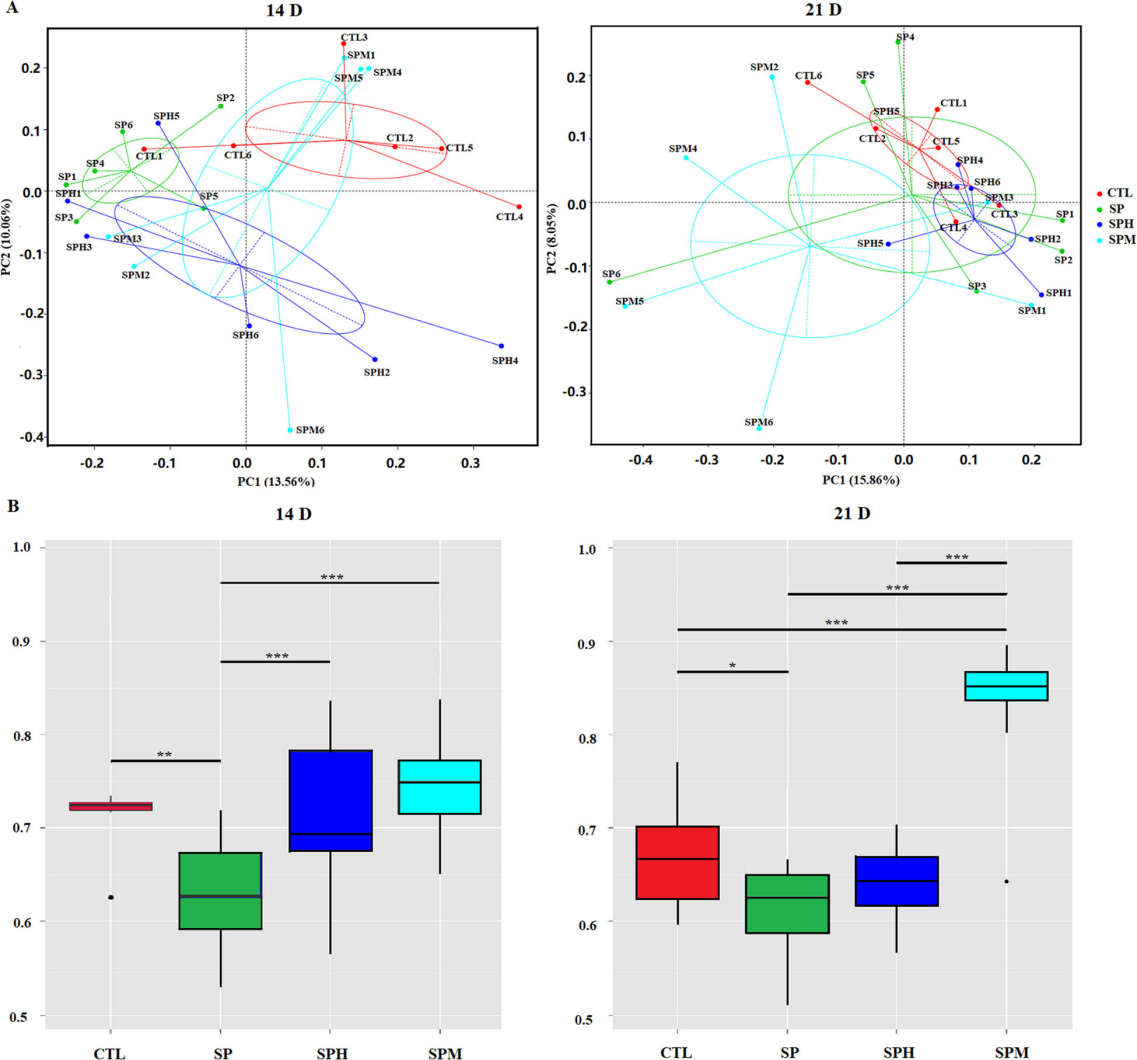
Beta diversity of the ileum microbiota of briolers. **A** Principal co-ordinates analysis (PCoA) plot of ileum microbiota according to the unweighted Unifrac distance metrics. **B** Beta diversity index of the ileum microbiota of briolers. Box plots show means and quartiles (*n* = 6); * means *P* < 0.05; ** means *P* < 0.01; *** means < 0.001

The β-diversity is shown in Fig. 2. The PCoA and boxplot of the β-diversity index based on unweighted UniFrac were performed to evaluate the differences in ileal microbial community structure among the four groups. At d 14, the PCoA plot of SPM was separated from the SP group. In addition, the β-diversity index of the SP group was significantly lower than that of the other groups (*P* < 0.01). At d 21, the PCoA plot showed that the ileal microbial community of the SPM group showed a trend of separation with the CTL and SPH groups. The β-diversity index of the SPM group was higher than that of the other groups (*P* < 0.001). In addition, this index was higher in the CTL group than in the SP group (*P* < 0.05).

**Fig. 3:**
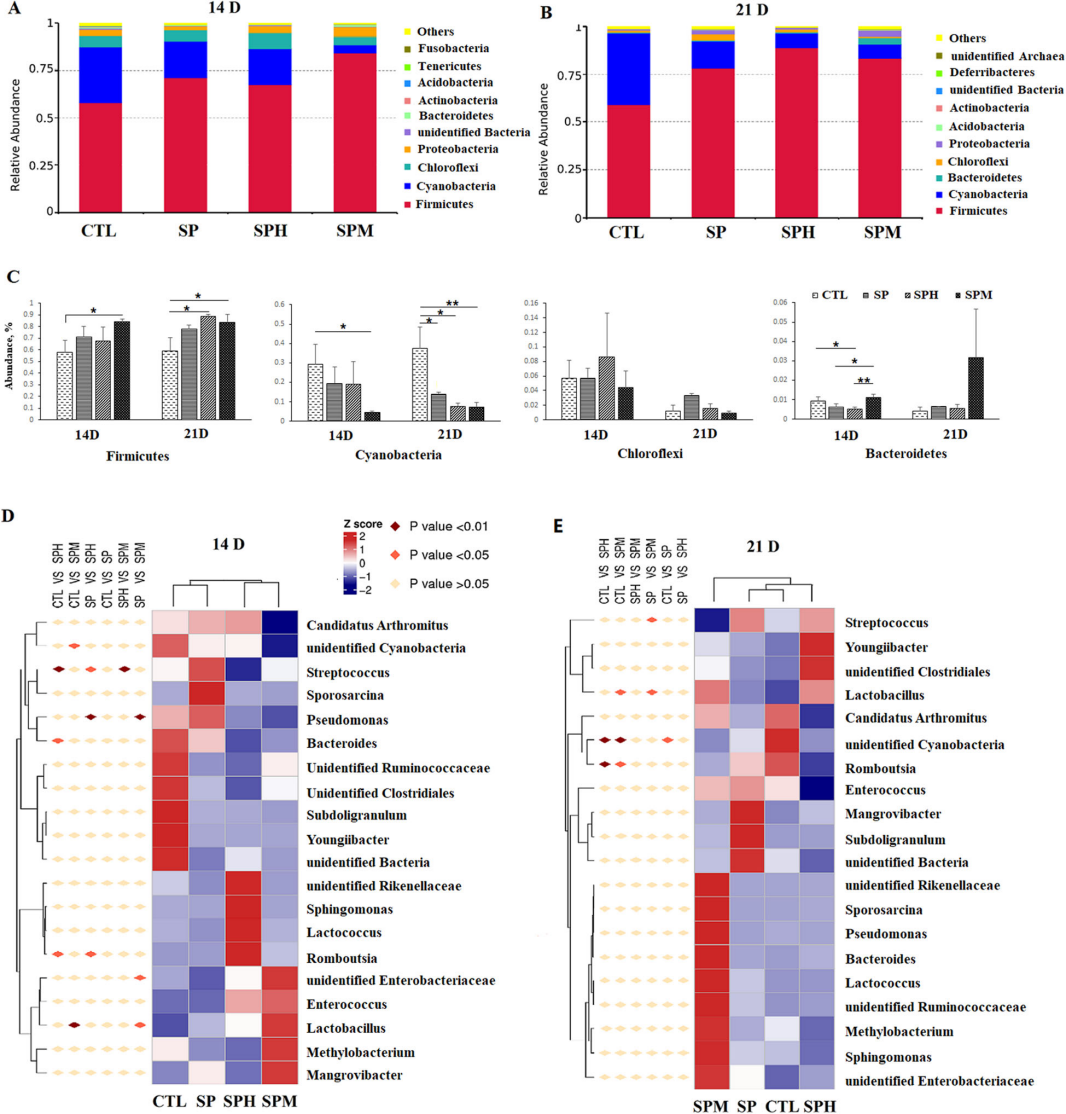
Ileum microbiota composition of briolers. **A** Gut microbiota composition of 14-day-old brioler at the phylum level. **B** Gut microbiota composition of 21-day-old brioler at the phylum level. **C** Different microbes at the phylum level. All values are expressed as the means ± SD (*n* = 6); * means *P* < 0.05; ** means *P* < 0.01; * means *P* < 0.05; ** means *P* < 0.01; **D** Different microbes at the genus level

At the phylum level (Fig. 3A, B, C), Firmicutes was the most dominant phylum in the ileum microbiota of 14-day-old and 21-day-old chickens, followed by Cyanobacteria. The relative abundance of Firmicutes was increased in the SPM group at 14 d (*P* < 0.05) and in the SPH and SPM groups at 21 d compared to the CTL group (*P* < 0.05). Meanwhile, the relative abundance of Cyanobacteria was decreased in the SPM group at 14 d (*P* < 0.05) and in all treatment groups at 21 d compared to the CTL group. In addition, the SP and SPH groups had a lower percentage of Bacteroidetes than the SPM group (*P* < 0.05). At the genus level (Fig. 3D, E), *Lactobacillus*, unidentified *Cyanobacteria*, *Streptococcus*, *Candidatus, Arthromitus*, and *Enterococcus* were the dominant bacteria in the ileum. At 14 d, magnolol supplementation increased the relative abundance of *Lactobacillus* (*P* < 0.01) compared to the CTL and SP groups, and decreased the abundance of unidentified *Cyanobacteria* (*P* < 0.05) compared to the CTL group. The relative abundance of unidentified *Enterobacteriaceae* in the SPM group was higher than in the SP group (*P* < 0.05). Honokiol supplementation increased the abundance of *Romboutsia* compared to the CTL and SP groups (*P* < 0.05), and there was a decreased amount of *Bacteroides* (*P* < 0.05) and *Streptococcus* (*P* < 0.01) compared to the CTL group. Both magnolol and honokiol decreased the abundance of *Pseudomonas* compared to the SP group (*P* < 0.01). At 21 d, magnolol supplementation increased the abundance of *Lactobacillus* compared to the CTL and SP groups, and decreased the abundance of *Streptococcus* compared to the SP group. Both magnolol and honokiol had a lower abundance of unidentified *Cyanobacteria* and *Romboutsia* than the CTL group.

**Fig. 4:**
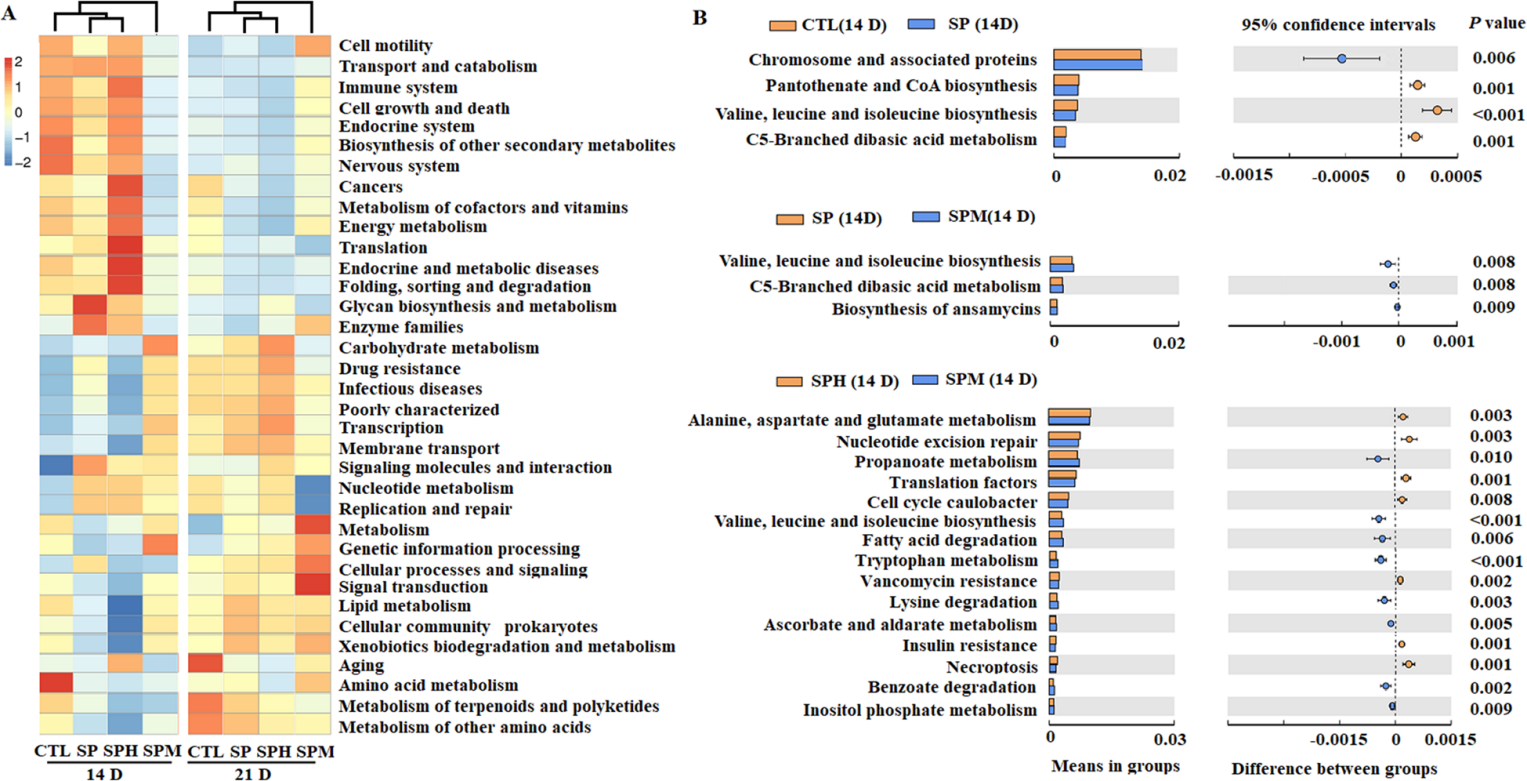
Predictive functional profiles of the ileum microbiota of briolers. **A** Predictive functional profiles at second level pathways. The top 35 pathways were list. **B** Predictive functional profiles at second level pathways. The pathways were list when *P* < 0.01

The changes in the presumptive functions of the ileum microbiota are shown in Fig. 4. At the second level of the KEGG pathways (Fig. 4A), the predictive functional profiles of the 16S rRNA marker genes from the ileum microbiota of the SP and SPH groups were classified into the same cluster, with the SPM group separated into one cluster with the other three groups at two time points. At the three level of the KEGG pathways (Fig. 4B), pathways related to pantothenate and CoA biosynthesis, valine, aspartate, and glutamate metabolism, and C5-Branched dibasic acid metabolism were decreased in the SP group compared to the CTL group at d 14 (*P* < 0.01). Conversely, pathways related to valine, leucine, and isoleucine biosynthesis metabolism, C5-Branched dibasic acid metabolism, and biosynthesis of ansamycins were higher in the SPM group than in the SP group at d 14 (*P* < 0.01). Compared to honokiol, magnolol could increase the number of microbial genes involved in propanoate metabolism, valine, aspartate, and glutamate metabolism, fatty acid and lysine degradation, tryptophan metabolism, ascorbate and aldarate metabolism, and inositol phosphate metabolism, and decreased the number of microbial genes involved in alanine, aspartate, and glutamate metabolism at d 14 (*P* < 0.01).

### Gene expression of the ileum

**Fig. 5:**
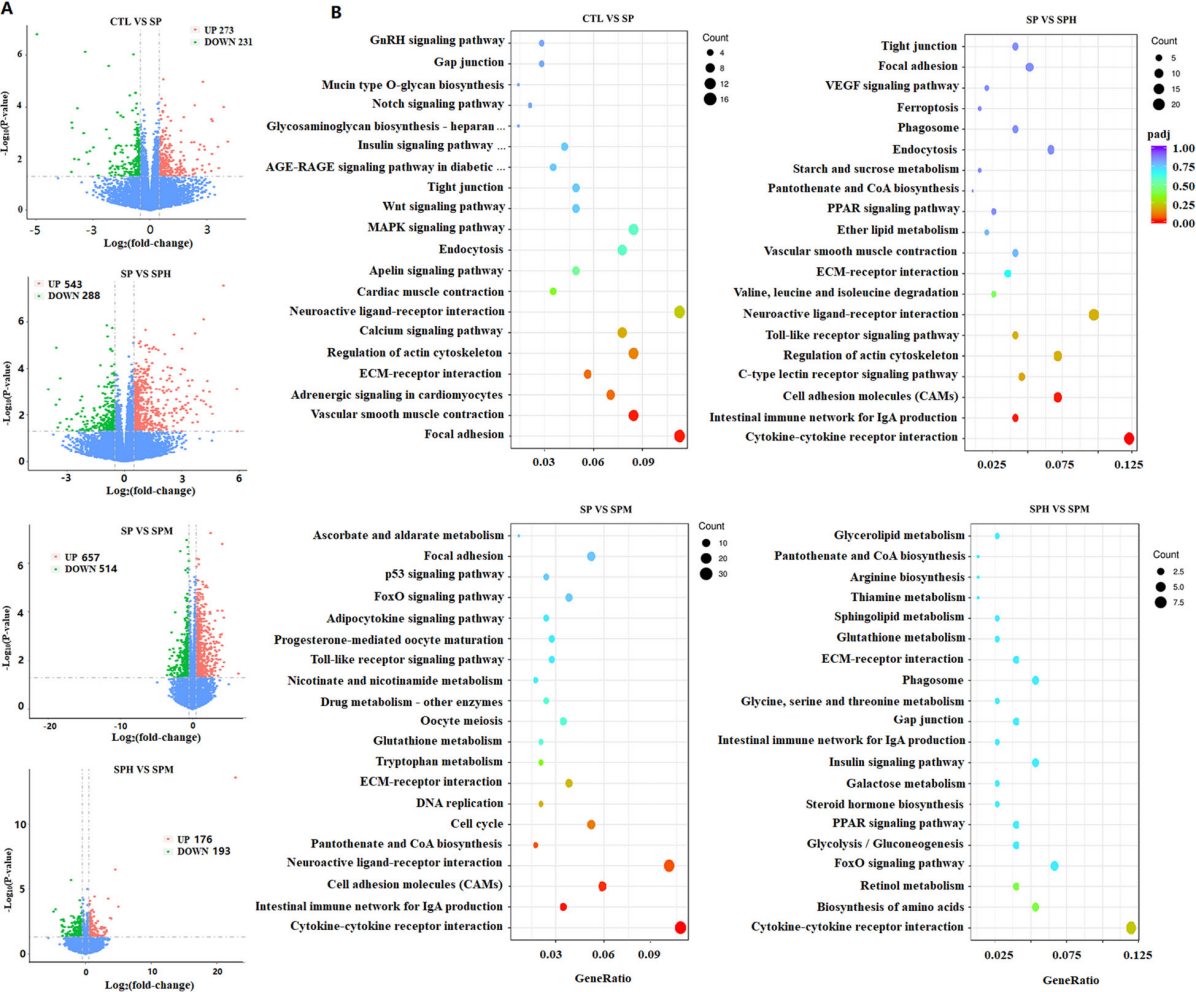
RNA-seq analysis of the ileum of briolers. **A** Volcano plot of differentially expressed genes. Red represents increased expression while green represents decreased expression. **B** Dot plot of KEGG pathway enrichment analyses of differentially expressed genes

**Fig. 6:**
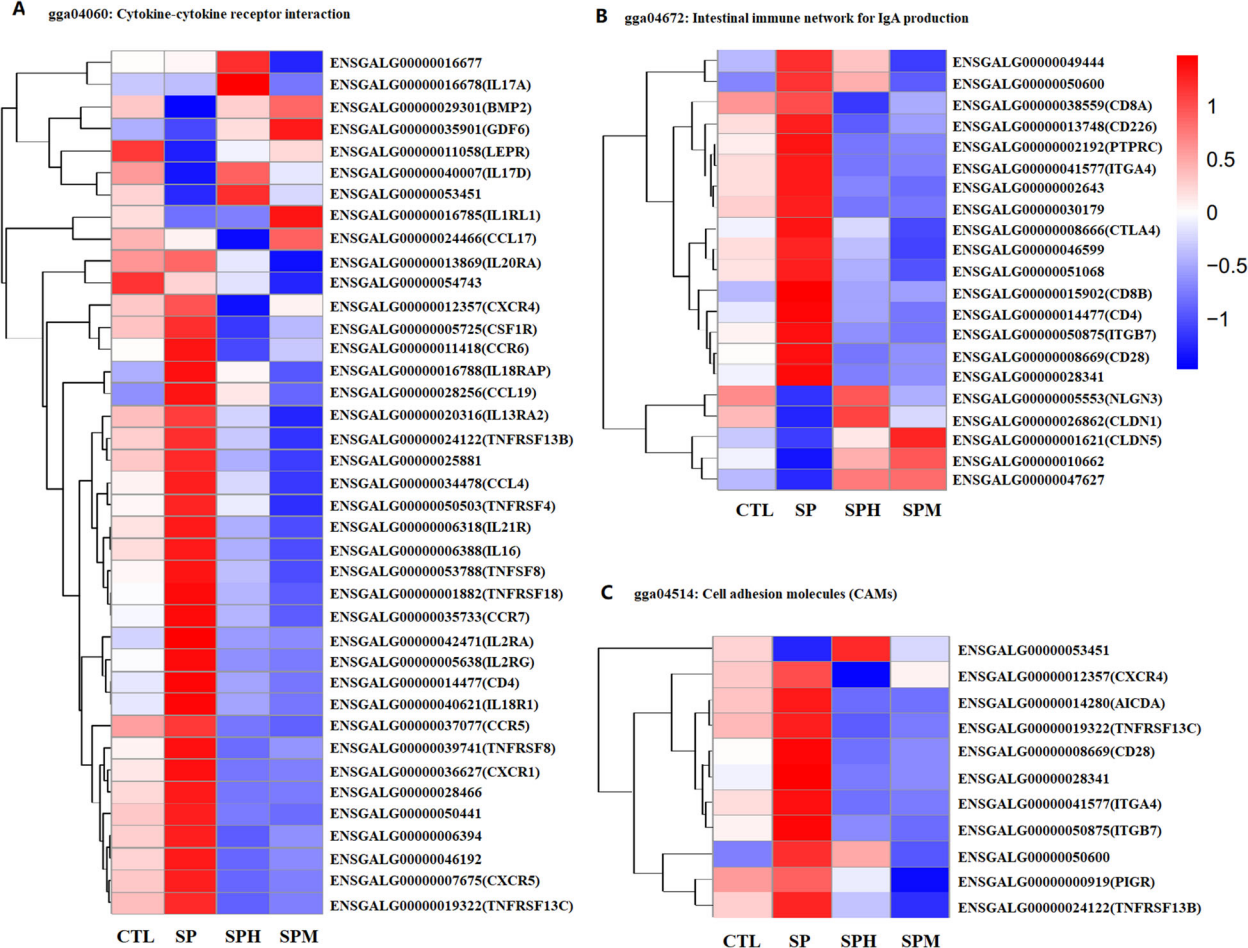
Heatmap of the differentially expressed gene in three specific KEGG pathways. **A** Heatmap representing differentially expressed genes enriched in KEGG pathway “cytokine-cytokine receptor interaction”. **B** Heatmap representing differentially expressed genes enriched in KEGG pathway “intestinal immune network for IgA production”. **C** Heatmap representing differentially expressed genes enriched in KEGG pathway “cell adhesion molecules (CAMs)”. Red represents increased expression while blue represents decreased expression

In the present study, we assessed the impact of magnolol and honokiol on ileum gene expression in broilers infected with Salmonella pullorum on d 14. Volcano plots of transcriptome sequencing data were used to visualize the distribution of differentially expressed genes (DEGs) between the two groups (Fig. 5A). The top 20 pathways associated with DEGs using a KEGG enrichment analysis are illustrated in Fig. 5B. Compared with CTL group, we found 504 DEGs, including 273 upregulated genes and 231 downregulated genes, in the SP group. The KEGG pathway analysis uncovered pathways that were significantly enriched with DEGs, including the focal adhesion and vascular smooth muscle contraction pathways. Compared with SP group, we observed 831 DEGs in the SPH group and 1,171 DEGs in SPM group. Notably, cytokine-cytokine receptor interactions, intestinal immune network for IgA production, and cell adhesion molecule pathways were the same pathways which is differentially regulated in the SPH and SPM groups (Fig. 6). Because cytokines play a crucial role in immune responses, we focused on the cytokine-cytokine receptor interactions pathway. Compared with uninfected group, *S. pullorum* infection increased the expression of *IL18RAP*, *CCL19*, *CCR7* and decrease the expression of *LEPR* and *IL17D*. Compared to the SP group, magnolol and honokiol supplementation could decease the expression of *CSF1R, IL21R, IL16, TNFSF8, TNFRSF18, CCR7, IL2RA, IL2RG, CD4, IL18R1, CCR5, TNFRSF8, CXCR1, CXCR5, TNFRSF13C*. In addition to these genes, magnolol could decrease the mRNA level of *CCL19*, *IL20RA, IL18RAP, IL13RA2, TNFRSF4, IL21R* and increase the expression of *BMP2, GDF6, LEPR, IL1RL1* while honokiol could decrease the mRNA level of *CCL17*, *CXCR4*, *CCR6* and increase *IL17A* and *IL17D* expression.

**Fig. 7:**
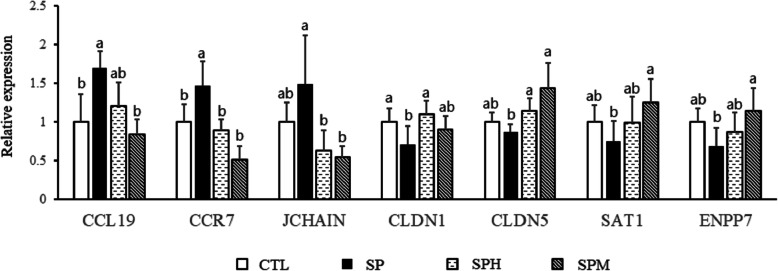
Relative expression levels from qRT-PCR. *CCL19*: chemokine ligand 19; *CCR7*: chemokine receptor 7; *JCHAIN*: joining chain of multimeric IgA and IgM; *CLDN1*: claudin 1; *CLDN5*, claudin 5; *SAT1*: spermidine-spermine acetyltransferase 1; *ENPP7*: alkaline sphingomyelinase; All values are expressed as the means ± SD (*n* = 6); Column witout same letters means significantly different (*P* < 0.05) among groups

We listed the top 100 DEGs according to their FPKM values (Additional file 3). We chose seven genes involved in nutrient metabolism (*SAT1*, *ENPP7*), immune (*CCL19*, *CCR7, JCHAIN*), tight junction proteins (*CLDN1*, *CLDN5*) to validate using RT-qPCR (Fig. 7). Compared with control group, *S. pullorum* infection increased the expression of *CCL19*, *CCR7* and decreased the expression of *CLDN1* (*P* < 0.05)*.* Compared with SP group, dietary magnolol and honokiol supplementation could decrease the expression of *CCR7*, *JCHAIN* and increase the expression of *CLDN5* (*P* < 0.05). In addition, magnolol could also increase the expression of *SAT1*, *ENPP7*, *CLDN5* and decrease the expression of *CCL19* while honokiol could also enhance the expression of *CLDN1* (*P* < 0.05). The results showed a good correlation between the RT-qPCR results and the RNA-seq data.

## Discussion

Diseases caused by *S. pullorum* in chickens, known as pullorum disease, pose a great threat to the poultry industry, mainly in developing countries [13, 14]. Salmonella infections resulting in compromised production performances, intestinal damage, and acute systemic diseases in broiler chickens have been widely reported [5, 15]. Young chicks were highly susceptible to *S. pullorum* within 20 d of age*.* In the present study, *S. pullorum* infection impaired the chicks’ growth, although mortality was not observed. Magnolol supplementation mitigated the compromised production performance, as indicated by the average weight, ADG, and decreased FCR. In terms of growth performance, magnolol had a better effect than honokiol in protecting chicks infected with *S. pullorum*.

However, the weakened virulence as a result of culturing for several generations did not lead to a high mortality, but did effectively activate the broilers’ immune systems. The bursa of Fabricius is a unique immune organ for B cell production in nestlings, which is essential for the adaptive immune response [16]. The spleen is a secondary lymphoid organ for both the innate and adaptive immune responses in chickens [17]. The relative weight of the bursa of Fabricius and spleen is a common indicator for evaluating the immune status of chickens [16]. In the present study, *S. pullorum* infection effectively activated the immune response, as indicated by the increased spleen and bursa of Fabricius weights. The magnolol and honokiol supplementation groups had spleen and bursa of Fabricius weights that were similar to the uninfected group. Meanwhile, the serum globulin level of the SP group was higher than that of the CTL and SPM groups at 14 d. Immunoglobulin, the major species of globulin in the serum, would significantly increase after *Salmonella* infection [18]. The intestine plays a crucial role in the mucosal immune system and provides antigen-specific protection [19]. *Salmonella pullorum* infection could induce marked intestinal immune responses [5]. Cytokines play a crucial role in immune responses by aiding cell-to-cell communication. *CCL19* and its receptor *CCR7*, *JCHAIN* were differential expression genes with relatively high expression in ileal. The CCL19-CCR7 axis is a critical regulator of adaptive immunity and inflammatory responses [20]. JCHAIN plays an important role in the secretion and transport of immunoglobulins and activation of complement [21]. Compared with control group, *CCL19*, *CCR7* were markedly overexpressed in S. pullorum infection group. Intriguingly, magnolol and honokiol supplementation resulted in lower levels of *CCL19*, *CCR7* and *JCHAIN* compared to the SP group. All these results suggest that the SP group remained in an immune reactive state, but the SPM and SPH groups seemed to recover from their *S. pullorum* infection at 14 d.

Intact intestinal mucosal structures are essential to maintain the health and production performance of animals. Villus heights, crypt depths, and villus/crypt ratios are important indices of the intact intestinal mucosal structure [22]. Some studies have reported that *S. pullorum* infections can decrease the height of the villus and villus/crypt ratio [5, 22]. Consistent with these reports, *S. pullorum* infections significantly reduced villus height in the jejunum and ileum, and this adverse effect lasted until at least 21 days of age. In our previous study, we confirmed that magnolol could increase the intestinal villus height of laying hens [23]. Magnolol has also been shown to improve the intestinal mucosal status of *Linwu* ducks [24]. Consistently, magnolol and honokiol effectively alleviated the damage to intestinal villi caused by *S. pullorum*. The genes claudin-1 (*CLDN1*) and claudin-5 (*CLDN5*), key component of tight junction protein genes, play important roles in gut barrier formation [25]. Reduced gene expression of tight junction proteins is usually associated with pathogenic challenges and pathological conditions [26]. It is worth noting that magnolol and honokiol upregulated the expression of *CLDN1* and *CLDN5* in this study. Remarkably, magnolol had a better effect than honokiol in increasing the height of the ileum villus at 21 d. Therefore, these different effects of magnolol and honokiol in defending against *S. pullorum* infection, to some extent, are related to improvements in the structure of the intestinal mucosal and to its barrier functions.

The intestinal microbiota plays a pivotal role in nutrient delivery and in the maintenance of multiple physiological processes that are related to host health. The diversity of the intestinal bacteria is beneficial for maintaining the stability of the bacteria and the environment in the intestines, as well as to resist the invasion of pathogenic bacteria [27]. Lower gut bacterial diversity is associated with poor health [28]. *S. pullorum* challenge has been suggested to result in intestinal health problems and disturbances to the gut microbiota in chickens [29]. Changing the composition and increasing the diversity of intestinal bacteria can be used as a strategy to resist the challenge of Salmonella and improve the growth performance of broiler chickens [30, 31]. Based on alpha diversity measurements, *S. pullorum* infections significantly reduced the community richness and phylogenetic diversity of intestinal bacteria of broiler at 14 d, as indicated by the reduced ACE estimator and PD whole tree index, respectively. Magnolol effectively increased the community richness and diversity of the bacterial community at 21 d, as indicated by the increased observed species, Chao, ACE, Shannon and PD whole tree index. In addition, the β-diversity analysis indicated a distinction in the SPM microbial community structure relative to SP group. These results explain magnolol’s improved effect at protecting broilers from *S. pullorum* infections.

Consistent with the results of previous studies, Firmicutes and Cyanobacteria were the most abundant in the ileum at the phylum level [30, 32]. Firmicutes play an important role in polysaccharide decomposition and contribute to the maintenance of intestinal homeostasis and health [33, 34]. Cyanobacteria are responsible for the production of some potential neurotoxic or pro-inflammatory activities [35]. A recent study reported that enrichment of Firmicutes and reductions of Cyanobacteria in the ileum that are caused by the addition of *Kluyveromyces marxianus* may be linked to the improved feed efficiency and intestinal structure of broilers [36]. In the present study, an increased abundance of phylum Firmicutes and a reduction of Cyanobacteria was observed in the magnolol addition group at 14 d; this was also seen in both the magnolol and honokiol groups at 21 d. Increased abundance of Bacteroides is related to high levels of fiber fermentation and volatile fatty acids, which contribute to reducing pathogen populations in the gut and promoting broiler growth [37, 38]. Notably, dietary supplementation with magnolol increased the abundance of the phylum Bacteroides compared to the SP group.

Consistent with other reports, *lactobacilli* are dominant in the ileum at the genus level [39, 40]. Several studies have repeatedly shown that *lactobacilli*, as the most important microbiota in animal intestines, have various biochemical functions, including in the protection of the integrity of the intestinal mucosal, the invasion of pathogens, and stimulation of the immune system [41, 42]. Many probiotic strains of *lactobacilli* have been widely used in poultry production [43]. The genus *Streptococcus* was highly abundant in the ileum microbiota of broiler chicks with necrotic enteritis associated with Salmonella challenge [44]. A study has shown that magnolol has antimicrobial abilities against some strains of the genus *Streptococcus* [45]. Our findings indicate that magnolol supplementation could increase the abundance of *Lactobacillu*s and decrease the abundance of *Streptococcus* and unidentified *Cyanobacteria* at the genus level. Remarkably, changes in these microbiotas were consistent at different detection time points. Except for these three genera, some genera showed different changes under different detection time points, such as *Pseudomonas*, *Bacteroides*, and *Romboutsia*. These results may be due to the weakened effects of Salmonella challenge in an increasing number of days.

The predicted functions of the ileum microbiota showed that pathways related to valine, aspartate, glutamate, and C5-branched dibasic acid metabolism were decreased in broilers challenged with *S. pullorum*, while magnolol could increase these pathways. Leucine, valine, and isoleucine, which are representative of branched-chain amino acids, have key physiological roles in the regulation of immunity [46] and fat metabolism [47]. Several studies have shown that C5-branched dibasic acid metabolism is related to energy generation and is enriched in healthy animals [48, 49]. In addition, magnolol addition increased the pathways related to the biosynthesis of ansamycins compared to the SP group. Rifamycins, as members of ansamycins, show antimicrobial activity against aerobic bacteria and Salmonellae [50]. Therefore, the predicted functions of the ileum microbiota have further proven the effectiveness of magnolol in improving intestinal flora.

Besides affecting gut metabolism by regulating flora composition, magnolol and honokiol had different effects on gut gene expression related to metabolism. Intestinal alkaline sphingomyelinase (ENPP7 or NPP7), an enzyme that hydrolyzes sphingomyelin to ceramide, can inhibit inflammation and stimulate cholesterol absorption [51]. Ceramide, the central point of the sphingolipid metabolic pathway, plays an important role in the innate immunity of intestinal epithelia against *Salmonella* infection [52]. SAT1 plays a crucial role in polyamine metabolism which is essential for gut mucosal growth and barrier function [53]. Magnolol increased the expression of *ENPP7* and *SAT1*, while honokiol had no significant effect on these genes. These results may partly explain that magnolol had better effect on alleviating *S. pullorum*-induced impairment in growth performance and intestinal villi structure. In order to better understand the mechanism of magnolol and honokiol, further studies are needed on the interaction between microorganisms and the gene expression of the host mucosa.

## Conclusions

In conclusion, supplemental magnolol and honokiol alleviated *S. pullorum-*induced impairment in growth performance and intestinal villi structure, and the effect of magnolol was better than that of honokiol, which could be partially due to magnolol’s ability to improve the composition of gut bacteria and improve the mucosal immune response. This study expands our fundamental knowledge concerning the role of gut microbiota in mediating the functional difference between magnolol and honokiol in broilers infected with *S. pullorum*.

## Supplementary Information

**Additional file 1.** Ingredients and nutrient levels of basal diets.

**Additional file 2.** Primers used in this study.

**Additional file 3.** The top 100 differentially expressed gene.

## Data Availability

The datasets of 16S rDNA gene sequencing of the ileum microbiome generated for this study can be found in the Sequence Read Archive (SRA) database (Bioproject ID: PRJNA688538). The datasets of RNA-Seq generated for this study can be found in the Gene Expression Omnibus (GEO) database: GSE164260.

## Abbreviations

BW: Body weight
FCR: Feed conversion
ADG: Average daily gain
ADFI: Average daily feed intake
TP: Total protein
A/G: Albumin/ globulin
AST: Aspartate aminotransferase
ALT: Alanine aminotransferase
ALP: Alkaline phosphatase
CCL19: Chemokine ligand 19
CCR7: Chemokine receptor 7
SAT1: Spermidine-spermine acetyltransferase 1
ENPP7: Alkaline sphingomyelinase
CLDN1: Claudin-1
CLDN5: Claudin-5
