# Supplementary material for: Supplemental magnolol or honokiol attenuates adverse effects in broilers infected with Salmonella pullorum by modulating mucosal gene expression and the gut microbiota
Source: J Anim Sci Biotechnol. 2021 Aug 9;12:87. doi: 10.1186/s40104-021-00611-0 (PMC8351427; doi:10.1186/s40104-021-00611-0)
Supplement: Supplementary file 1 — Additional file 1. Ingredients and nutrient levels of basal diets. [file 40104_2021_611_MOESM1_ESM.pdf]

Supplementary Material

Table S1. Ingredients and nutrient levels of basal diets (%, as air-dry basis)

Ingredient		Nutrient composition	
Maize	55.53	Metabolic energy (MJ/kg)	12.10
Soybean meal	37.6	Crude protein	20.50
Soybean oil	2.50	Lysine	1.15
Dicalcium phosphate	1.90	Methionine	0.50
Limestone	1.24	Threonine	0.81
Salt	0.35	Calcium	1.01
Choline chloride (50%)	0.30	Available phosphorus	0.41
DL-methionine (98%)	0.21		
L-lysine (78%)	0.08		
L-threonine (98.5%)	0.04		
Premix	0.25		
Total	100		

The premix provided the following (per kilogram of diet): Iron, 80 mg; Copper, 8 mg; Zinc, 75 mg; Manganese, 100 mg; Selenium, 0.15 mg; Iodine, 0.35 mg; Vitamin A, 10,000 IU; Vitamin D3, 2,400 IU; Vitamin E, 40 IU; Vitamin K_3, 2.5 mg; Niacin, 30 mg; Pantothenate 8 mg; Folacin, 1 mg; Vitamin B_1, 2.5 mg; Vitamin B_2, 5.5 mg; Vitamin B_6, 4 mg; Vitamin B_{12}, 20 µg. Metabolic energy was calculated value while other nutrient levels were measured values.
