# Supplementary material for: Supplemental magnolol or honokiol attenuates adverse effects in broilers infected with Salmonella pullorum by modulating mucosal gene expression and the gut microbiota
Source: J Anim Sci Biotechnol. 2021 Aug 9;12:87. doi: 10.1186/s40104-021-00611-0 (PMC8351427; doi:10.1186/s40104-021-00611-0)
Supplement: Supplementary file 2 — Additional file 2. Primers used in this study. [file 40104_2021_611_MOESM2_ESM.pdf]

Supplementary Material

Additional file 2. The primers used in this study

Primer name	Primer sequence (5'-3')	Annealing temperature, ℃	Accession No
CCL19 F	GACTGCTGCCTGCGGACGA	60	NM_001302168
CCL19 R	GCAAAGGGCAAGCGTCTCTG		
CCR7 F	CCGACGACTATGACGCCAAC	59	NM_001198752
CCR7 R	GCCAGGTTCAGCAAGTAGATG		
JCHAIN F	CTACCGCATGACTGAACTCTG	59	NM_204263.1
JCHAIN R	GTGTAGCATTTGTCCCTGTTG		
ENPP7 F	GATACGAAGTCCTGGCTGAAG	58	XM_015295596.2
ENPP7 R	CTACAGGGAGGGCAGCAAAC		
SAT1 F	GAGAGAAGGTTCCAGCGACT	58	NM_204186.1
SAT1 R	CAAAGATTACAAGCCCACACT		
CLDN1 F	CTGATTGCTTCCAACCAG	58	NM_001013611.2
CLDN1 R	CAGGTCAAACAGAGGTACAGG		
CLDN5 F	CATCACTTCTCCTTCGTCAGC	59	NM_204201.1
CLDN5 R	GCACAAAGATCTCCCAGGTC		
β-actin F	GAGAAATTGTGCGTGACATCA	58	NM_205518.1
β-actin R	CCTGAACCTCTCATTGCCA		

CCL19: chemokine ligand 19; CCR7: chemokine receptor 7; JCHAIN: joining chain of multimeric IgA and IgM; SAT1: spermidine-spermine acetyltransferase 1; ENPP7: alkaline sphingomyelinase; CLDN1: claudin-1; CLDN5: claudin
