# Supplementary material for: Supplemental magnolol or honokiol attenuates adverse effects in broilers infected with Salmonella pullorum by modulating mucosal gene expression and the gut microbiota
Source: J Anim Sci Biotechnol. 2021 Aug 9;12:87. doi: 10.1186/s40104-021-00611-0 (PMC8351427; doi:10.1186/s40104-021-00611-0)
Supplement: Supplementary file 3 — Additional file 3. The top 100 differentially expressed gene. [file 40104_2021_611_MOESM3_ESM.pdf]

Supplementary Material

Additional file 3. The top 100 differentially expressed gene

Gene id	Gene name	CTL_FPKM	SP_FPKM	SPH_FPKM	SPM_FPKM	CTLvsSP_log$_2$FoldChange	CTLvsSP_Pvalue	CTLvsSPH_log$_2$FoldChange	CTLvsSPH_Pvalue	CTLvsSPM_log$_2$FoldChange	CTLvsSPM_Pvalue	SPvsSPM_log$_2$FoldChange	SPvsSPM_Pvalue	SPvsSPH_log$_2$FoldChange	SPvsSPH_Pvalue	SPMvsSPH_log$_2$FoldChange	SPMvsSPH_Pvalue
ENSGALG00000014176	PLA2G2E	4981.49	3589.55	5218.76	3704.85	0.47	0.04	-0.07	0.70	0.43	0.03	-0.05	0.84	-0.54	0.02	-0.50	0.01
ENSGALG00000041502	ANGPT1L	3627.10	3362.21	4659.45	2716.86	0.11	0.81	-0.36	0.32	0.42	0.29	0.31	0.43	-0.47	0.19	-0.78	0.01
ENSGALG00000001445	FABP6	2596.10	3568.92	2394.31	3027.87	-0.46	0.04	0.12	0.58	-0.22	0.40	0.24	0.36	0.57	0.00	0.34	0.16
ENSGALG00000042750	ND1	1491.39	1358.88	1471.30	1966.83	0.13	0.37	0.02	0.90	-0.40	0.01	-0.53	0.00	-0.12	0.46	0.42	0.01
ENSGALG00000016348	SAT1	1079.32	1001.42	1033.45	1491.06	0.11	0.68	0.06	0.78	-0.47	0.06	-0.58	0.01	-0.05	0.82	0.53	0.00
ENSGALG00000010825	AGR2	1004.77	1183.90	1529.82	983.10	-0.24	0.34	-0.61	0.00	0.03	0.89	0.27	0.33	-0.37	0.12	-0.64	0.01

Gene id	Gene name	CTL_FPKM	SP_FPKM	SPH_FPKM	SPM_FPKM	CTLvsSP_log$_2$FoldChange	CTLvsSP_Pvalue	CTLvsSPH_log$_2$FoldChange	CTLvsSPH_Pvalue	CTLvsSPM_log$_2$FoldChange	CTLvsSPM_Pvalue	SPvsSPM_log$_2$FoldChange	SPvsSPM_Pvalue	SPvsSPH_log$_2$FoldChange	SPvsSPH_Pvalue	SPMvsSPH_log$_2$FoldChange	SPMvsSPH_Pvalue
ENSGALG00000033033	MEP1A	736.84	591.06	601.89	1107.64	0.31	0.32	0.29	0.40	-0.59	0.02	-0.91	0.00	-0.03	0.95	0.88	0.00
ENSGALG00000015082	RPS6	706.50	1034.37	990.17	868.93	-0.55	0.00	-0.49	0.01	-0.30	0.09	0.25	0.28	0.06	0.80	-0.19	0.42
ENSGALG00000013743	ENPP7	643.83	566.55	550.43	1010.49	0.18	0.51	0.23	0.27	-0.65	0.00	-0.84	0.00	0.04	0.88	0.87	0.00
ENSGALG00000015617	RPS23	596.59	892.73	855.45	758.22	-0.58	0.00	-0.52	0.02	-0.34	0.05	0.23	0.30	0.06	0.82	-0.18	0.50
ENSGALG00000010769	HPGD	482.09	373.34	395.85	588.27	0.37	0.18	0.28	0.23	-0.29	0.23	-0.66	0.02	-0.09	0.76	0.57	0.02
ENSGALG00000004262	TMIGD1	396.84	416.90	447.12	587.75	-0.07	0.66	-0.17	0.27	-0.56	0.00	-0.50	0.01	-0.10	0.56	0.39	0.02
ENSGALG00000000919	PIGR	383.10	402.46	345.24	282.94	-0.07	0.82	0.15	0.72	0.44	0.16	0.51	0.03	0.22	0.46	-0.29	0.31

Gene id	Gene name	CTL_FPKM	SP_FPKM	SPH_FPKM	SPM_FPKM	CTLvsSP_log$_2$FoldChange	CTLvsSP_Pvalue	CTLvsSPH_log$_2$FoldChange	CTLvsSPH_Pvalue	CTLvsSPM_log$_2$FoldChange	CTLvsSPM_Pvalue	SPvsSPM_log$_2$FoldChange	SPvsSPM_Pvalue	SPvsSPH_log$_2$FoldChange	SPvsSPH_Pvalue	SPMvsSPH_log$_2$FoldChange	SPMvsSPH_Pvalue
ENSGALG00000004449	CYP3A5	355.67	353.68	305.87	438.52	0.01	0.98	0.22	0.36	-0.30	0.26	-0.31	0.28	0.21	0.42	0.52	0.03
ENSGALG00000026970	IFITM3	308.99	449.34	292.70	335.80	-0.54	0.01	0.08	0.64	-0.12	0.53	0.42	0.08	0.62	0.01	0.20	0.34
ENSGALG00000019527	SLC6A19	300.67	381.33	333.38	441.28	-0.35	0.12	-0.15	0.39	-0.55	0.01	-0.21	0.41	0.19	0.38	0.40	0.07
ENSGALG00000015704	TXN	296.97	415.88	458.98	396.93	-0.49	0.01	-0.63	0.01	-0.42	0.01	0.07	0.77	-0.14	0.61	-0.21	0.40
ENSGALG00000032465	ATP8	286.41	248.12	299.95	400.03	0.20	0.36	-0.07	0.73	-0.48	0.03	-0.69	0.00	-0.27	0.09	0.41	0.01
ENSGALG00000028451	MT4L	273.76	159.80	168.10	207.11	0.77	0.01	0.70	0.02	0.40	0.14	-0.38	0.13	-0.07	0.78	0.30	0.17
ENSGALG00000013548	GZMA	237.97	281.38	400.26	328.60	-0.24	0.42	-0.75	0.03	-0.46	0.15	-0.22	0.52	-0.51	0.11	-0.29	0.44

Gene id	Gene name	CTL_FPKM	SP_FPKM	SPH_FPKM	SPM_FPKM	CTLvsSP_log2FoldChange	CTLvsSP_Pvalue	CTLvsSPH_log2FoldChange	CTLvsSPH_Pvalue	CTLvsSPM_log2FoldChange	CTLvsSPM_Pvalue	SPvsSPM_log2FoldChange	SPvsSPM_Pvalue	SPvsSPH_log2FoldChange	SPvsSPH_Pvalue	SPMvsSPH_log2FoldChange	SPMvsSPH_Pvalue
ENSGALG00000049414	CYP2C23a	224.68	191.38	178.79	312.47	0.23	0.47	0.33	0.18	-0.47	0.09	-0.71	0.05	0.10	0.80	0.80	0.01
ENSGALG00000002792	SPDEF	215.28	208.19	219.73	153.37	0.05	0.79	-0.03	0.82	0.49	0.03	0.44	0.08	-0.08	0.66	-0.52	0.02
ENSGALG00000008516	CPO	201.84	241.39	185.95	322.75	-0.26	0.44	0.12	0.79	-0.67	0.01	-0.42	0.17	0.38	0.43	0.79	0.03
ENSGALG00000033694	IFI30	200.95	224.73	154.75	174.32	-0.16	0.55	0.38	0.07	0.21	0.35	0.36	0.12	0.54	0.01	0.17	0.25
ENSGALG00000043234	HBA1	192.69	188.90	232.47	358.91	0.03	0.95	-0.27	0.59	-0.90	0.08	-0.93	0.01	-0.30	0.30	0.62	0.12
ENSGALG00000027963	COX7C	190.02	296.47	324.58	269.53	-0.64	0.00	-0.77	0.01	-0.50	0.01	0.14	0.61	-0.13	0.69	-0.27	0.49
ENSGALG00000011551	JCHAIN	184.16	227.21	125.48	95.07	-0.30	0.59	0.55	0.34	0.96	0.00	1.26	0.02	0.85	0.25	-0.40	0.49

Gene id	Gene name	CTL_FPKM	SP_FPKM	SPH_FPKM	SPM_FPKM	CTLvsSP_log$_2$FoldChange	CTLvsSP_Pvalue	CTLvsSPH_log$_2$FoldChange	CTLvsSPH_Pvalue	CTLvsSPM_log$_2$FoldChange	CTLvsSPM_Pvalue	SPvsSPM_log$_2$FoldChange	SPvsSPM_Pvalue	SPvsSPH_log$_2$FoldChange	SPvsSPH_Pvalue	SPMvsSPH_log$_2$FoldChange	SPMvsSPH_Pvalue
ENSGALG00000015372	ATP5ME	182.79	291.72	338.72	282.81	-0.68	0.00	-0.89	0.00	-0.63	0.00	0.04	0.88	-0.22	0.50	-0.26	0.50
ENSGALG00000037880	ATP5A1Z	182.69	282.07	286.84	243.20	-0.63	0.01	-0.65	0.01	-0.41	0.02	0.21	0.42	-0.02	0.94	-0.24	0.37
ENSGALG00000004196	UGT1A1	173.31	134.03	154.53	190.08	0.37	0.07	0.16	0.36	-0.13	0.45	-0.51	0.02	-0.21	0.35	0.30	0.13
ENSGALG00000027483	GLRX	164.45	211.78	252.24	194.29	-0.37	0.12	-0.62	0.01	-0.24	0.29	0.12	0.64	-0.25	0.37	-0.38	0.16
ENSGALG00000013723	OASL	156.49	212.02	145.78	183.48	-0.44	0.08	0.10	0.70	-0.23	0.30	0.21	0.31	0.54	0.03	0.33	0.13
ENSGALG00000053860	LOC420486	139.44	139.61	202.26	147.16	0.00	0.99	-0.54	0.02	-0.08	0.79	-0.08	0.87	-0.54	0.21	-0.46	0.06
ENSGALG00000031408	SORD	135.10	129.73	133.10	187.89	0.06	0.80	0.02	0.92	-0.47	0.02	-0.54	0.01	-0.04	0.86	0.50	0.01

Gene id	Gene name	CTL_FPKM	SP_FPKM	SPH_FPKM	SPM_FPKM	CTLvsSP_log$_2$FoldChange	CTLvsSP_Pvalue	CTLvsSPH_log$_2$FoldChange	CTLvsSPH_Pvalue	CTLvsSPM_log$_2$FoldChange	CTLvsSPM_Pvalue	SPvsSPM_log$_2$FoldChange	SPvsSPM_Pvalue	SPvsSPH_log$_2$FoldChange	SPvsSPH_Pvalue	SPMvsSPH_log$_2$FoldChange	SPMvsSPH_Pvalue
ENSGALG00000041621	LY6E	129.25	214.14	126.80	140.03	-0.73	0.00	0.03	0.92	-0.11	0.64	0.61	0.01	0.75	0.00	0.14	0.61
ENSGALG00000030187	CAT	127.47	110.32	124.60	160.96	0.21	0.34	0.03	0.87	-0.33	0.08	-0.55	0.01	-0.18	0.42	0.37	0.05
ENSGALG00000051301	KHK	122.66	87.38	145.68	147.01	0.49	0.03	-0.25	0.20	-0.26	0.25	-0.75	0.00	-0.74	0.00	0.01	0.95
ENSGALG00000044606	MEP1B	120.36	110.11	118.31	168.84	0.13	0.64	0.02	0.91	-0.49	0.07	-0.62	0.04	-0.10	0.69	0.51	0.04
ENSGALG00000013535	CYP4V2	119.09	111.78	110.08	161.78	0.09	0.78	0.11	0.64	-0.44	0.05	-0.53	0.10	0.02	0.96	0.55	0.03
ENSGALG00000050515	LOC107051274	113.29	355.61	94.97	63.97	-1.65	0.04	0.26	0.78	0.83	0.26	2.47	0.00	1.90	0.04	-0.57	0.52
ENSGALG00000009973	SLC3A1	110.46	114.29	129.77	164.11	-0.05	0.75	-0.23	0.09	-0.57	0.00	-0.52	0.00	-0.18	0.30	0.34	0.02

Gene id	Gene name	CTL_FPKM	SP_FPKM	SPH_FPKM	SPM_FPKM	CTLvsSP_log$_2$FoldChange	CTLvsSP_Pvalue	CTLvsSPH_log$_2$FoldChange	CTLvsSPH_Pvalue	CTLvsSPM_log$_2$FoldChange	CTLvsSPM_Pvalue	SPvsSPM_log$_2$FoldChange	SPvsSPM_Pvalue	SPvsSPH_log$_2$FoldChange	SPvsSPH_Pvalue	SPMvsSPH_log$_2$FoldChange	SPMvsSPH_Pvalue
ENSGALG00000047890	PCP4	107.18	92.97	107.19	134.13	0.20	0.22	0.00	0.99	-0.32	0.22	-0.53	0.04	-0.21	0.17	0.32	0.20
ENSGALG00000006855	MELTF	106.63	91.45	101.77	132.54	0.22	0.39	0.07	0.73	-0.31	0.11	-0.54	0.01	-0.15	0.46	0.38	0.00
ENSGALG00000000162	DMB1	105.94	76.75	118.35	95.47	0.46	0.05	-0.16	0.52	0.15	0.46	-0.32	0.03	-0.63	0.00	-0.31	0.10
ENSGALG00000030969	CLEC3B	97.21	83.83	84.77	121.13	0.21	0.20	0.20	0.25	-0.32	0.12	-0.53	0.01	-0.02	0.91	0.51	0.01
ENSGALG00000038636	LAPTM5	96.41	121.80	79.92	81.56	-0.34	0.11	0.27	0.15	0.24	0.17	0.58	0.00	0.61	0.00	0.03	0.85
ENSGALG00000037854	TKFC	96.24	73.40	110.63	108.55	0.39	0.09	-0.20	0.21	-0.17	0.41	-0.57	0.02	-0.59	0.00	-0.03	0.86
ENSGALG00000012260	LCT	95.73	95.30	88.73	130.34	0.00	0.99	0.11	0.66	-0.44	0.07	-0.45	0.13	0.10	0.73	0.55	0.01

Gene id	Gene name	CTL_FPKM	SP_FPKM	SPH_FPKM	SPM_FPKM	CTLvsSP_log$_2$FoldChange	CTLvsSP_Pvalue	CTLvsSPH_log$_2$FoldChange	CTLvsSPH_Pvalue	CTLvsSPM_log$_2$FoldChange	CTLvsSPM_Pvalue	SPvsSPM_log$_2$FoldChange	SPvsSPM_Pvalue	SPvsSPH_log$_2$FoldChange	SPvsSPH_Pvalue	SPMvsSPH_log$_2$FoldChange	SPMvsSPH_Pvalue
ENSGALG00000015326	CLTA	91.88	138.75	147.02	118.64	-0.60	0.00	-0.68	0.00	-0.37	0.05	0.22	0.37	-0.08	0.76	-0.31	0.23
ENSGALG00000034218	PA2G4	91.58	107.78	92.31	73.48	-0.24	0.02	-0.01	0.90	0.32	0.00	0.55	0.00	0.22	0.02	-0.33	0.00
ENSGALG00000034358	RETSAT	90.88	84.67	114.80	76.55	0.10	0.61	-0.34	0.08	0.25	0.17	0.14	0.35	-0.44	0.01	-0.59	0.00
ENSGALG00000005849	PPDPF	90.80	67.41	96.29	77.58	0.43	0.03	-0.09	0.71	0.23	0.23	-0.20	0.13	-0.52	0.01	-0.31	0.10
ENSGALG00000005933	SLC5A11	90.54	71.80	113.25	102.53	0.33	0.02	-0.32	0.09	-0.18	0.25	-0.52	0.00	-0.66	0.00	-0.15	0.48
ENSGALG00000016986	LCP1	86.16	113.52	72.97	73.06	-0.40	0.04	0.24	0.19	0.24	0.18	0.63	0.00	0.64	0.00	0.00	1.00
ENSGALG00000032687	PHLDA2	83.11	92.00	75.06	63.70	-0.15	0.47	0.15	0.47	0.39	0.03	0.53	0.00	0.29	0.14	-0.24	0.16

Gene id	Gene name	CTL_FPKM	SP_FPKM	SPH_FPKM	SPM_FPKM	CTLvsSP_log$_2$FoldChange	CTLvsSP_Pvalue	CTLvsSPH_log$_2$FoldChange	CTLvsSPH_Pvalue	CTLvsSPM_log$_2$FoldChange	CTLvsSPM_Pvalue	SPvsSPM_log$_2$FoldChange	SPvsSPM_Pvalue	SPvsSPH_log$_2$FoldChange	SPvsSPH_Pvalue	SPMvsSPH_log$_2$FoldChange	SPMvsSPH_Pvalue
ENSGALG00000026152	GBP4L	82.18	80.58	96.84	122.26	0.03	0.92	-0.24	0.39	-0.57	0.07	-0.60	0.04	-0.27	0.28	0.33	0.24
ENSGALG00000032588	ARPC1B	82.13	102.93	70.42	69.43	-0.33	0.13	0.22	0.25	0.25	0.18	0.57	0.00	0.55	0.01	-0.02	0.89
ENSGALG00000037332	IGFBP7	80.30	70.21	89.45	107.93	0.19	0.13	-0.16	0.19	-0.42	0.01	-0.62	0.00	-0.35	0.00	0.27	0.08
ENSGALG00000014942	NSA2	79.51	118.33	101.30	96.75	-0.58	0.00	-0.35	0.09	-0.28	0.14	0.29	0.23	0.22	0.37	-0.07	0.78
ENSGALG00000002466	SLC2A5	77.51	61.82	84.45	95.71	0.32	0.20	-0.12	0.49	-0.30	0.19	-0.63	0.04	-0.45	0.09	0.18	0.47
ENSGALG00000011455	CL2	75.57	88.08	112.47	117.16	-0.22	0.38	-0.58	0.00	-0.63	0.01	-0.41	0.15	-0.35	0.16	0.06	0.80
ENSGALG00000006583	LSP1	74.20	97.79	62.89	64.03	-0.40	0.07	0.24	0.22	0.22	0.27	0.61	0.00	0.63	0.00	0.02	0.87

Gene id	Gene name	CTL_FPKM	SP_FPKM	SPH_FPKM	SPM_FPKM	CTLvsSP_log₂FoldChange	CTLvsSP_Pvalue	CTLvsSPH_log₂FoldChange	CTLvsSPH_Pvalue	CTLvsSPM_log₂FoldChange	CTLvsSPM_Pvalue	SPvsSPM_log₂FoldChange	SPvsSPM_Pvalue	SPvsSPH_log₂FoldChange	SPvsSPH_Pvalue	SPMvsSPH_log₂FoldChange	SPMvsSPH_Pvalue
ENSGALG00000036190	AOC1	74.10	74.28	76.74	108.08	-0.01	0.98	-0.05	0.80	-0.54	0.01	-0.54	0.03	-0.05	0.84	0.49	0.01
ENSGALG00000023689	ASS1	70.22	107.58	103.59	73.23	-0.62	0.03	-0.56	0.12	-0.06	0.82	0.55	0.06	0.05	0.90	-0.50	0.11
ENSGALG00000031597	HBAD	67.35	61.77	81.37	129.52	0.12	0.79	-0.27	0.59	-0.94	0.08	-1.07	0.00	-0.40	0.18	0.67	0.12
ENSGALG00000050831	LOC769704	67.33	71.39	99.50	121.75	-0.09	0.67	-0.56	0.01	-0.85	0.00	-0.77	0.00	-0.48	0.04	0.29	0.22
ENSGALG00000009639	DDX60	66.90	70.69	58.87	92.95	-0.08	0.76	0.18	0.42	-0.47	0.00	-0.40	0.18	0.26	0.50	0.66	0.01
ENSGALG00000003751	ASAH2	66.44	64.27	62.27	95.89	0.05	0.88	0.09	0.63	-0.53	0.01	-0.58	0.04	0.05	0.87	0.62	0.00
ENSGALG00000031331	PKIG	65.63	88.71	61.08	48.99	-0.44	0.06	0.10	0.56	0.43	0.02	0.86	0.00	0.54	0.01	-0.32	0.02

Gene id	Gene name	CTL_FPKM	SP_FPKM	SPH_FPKM	SPM_FPKM	CTLvsSP_log_2FoldChange	CTLvsSP_Pvalue	CTLvsSPH_log_2FoldChange	CTLvsSPH_Pvalue	CTLvsSPM_log_2FoldChange	CTLvsSPM_Pvalue	SPvsSPM_log_2FoldChange	SPvsSPM_Pvalue	SPvsSPH_log_2FoldChange	SPvsSPH_Pvalue	SPMvsSPH_log_2FoldChange	SPMvsSPH_Pvalue
ENSGALG00000004503	CENPV	64.44	69.01	63.11	92.81	-0.10	0.59	0.03	0.86	-0.53	0.01	-0.43	0.04	0.13	0.50	0.55	0.00
ENSGALG00000049652	LOC769668	62.69	40.73	37.08	40.08	0.62	0.02	0.76	0.00	0.65	0.00	0.02	0.92	0.13	0.56	0.11	0.51
ENSGALG00000017244	PRSS23	60.74	49.69	71.69	63.53	0.29	0.20	-0.24	0.24	-0.06	0.73	-0.36	0.04	-0.53	0.01	-0.18	0.24
ENSGALG00000014585	CCL26	60.11	100.41	96.45	49.87	-0.74	0.12	-0.68	0.23	0.27	0.40	1.01	0.03	0.06	0.93	-0.95	0.09
ENSGALG00000003085	CDK1	59.66	73.28	64.41	48.14	-0.30	0.09	-0.11	0.49	0.31	0.07	0.60	0.00	0.18	0.24	-0.42	0.01
ENSGALG00000007651	STAT1	59.16	81.64	56.46	58.50	-0.47	0.00	0.07	0.56	0.02	0.89	0.48	0.00	0.53	0.00	0.05	0.73
ENSGALG00000034960	MLXIPL	58.70	52.79	75.41	76.01	0.15	0.46	-0.36	0.00	-0.37	0.01	-0.53	0.01	-0.52	0.01	0.01	0.94

Gene id	Gene name	CTL_FPKM	SP_FPKM	SPH_FPKM	SPM_FPKM	CTLvsSP_log2FoldChange	CTLvsSP_Pvalue	CTLvsSPH_log2FoldChange	CTLvsSPH_Pvalue	CTLvsSPM_log2FoldChange	CTLvsSPM_Pvalue	SPvsSPM_log2FoldChange	SPvsSPM_Pvalue	SPvsSPH_log2FoldChange	SPvsSPH_Pvalue	SPMvsSPH_log2FoldChange	SPMvsSPH_Pvalue
ENSGALG00000045085	IFIT5	58.11	89.70	48.41	73.77	-0.63	0.03	0.26	0.47	-0.34	0.21	0.28	0.32	0.89	0.00	0.61	0.04
ENSGALG00000001986	VCP	58.08	84.35	81.35	68.42	-0.54	0.01	-0.49	0.02	-0.23	0.12	0.30	0.18	0.05	0.84	-0.25	0.25
ENSGALG00000012834	AKR1D1	57.94	71.82	97.64	110.51	-0.31	0.50	-0.75	0.18	-0.93	0.05	-0.62	0.04	-0.44	0.35	0.18	0.73
ENSGALG00000008780	CTBS	57.31	46.30	46.05	70.18	0.31	0.23	0.31	0.23	-0.29	0.18	-0.60	0.02	0.01	0.98	0.61	0.02
ENSGALG00000011391	AMN	57.27	61.73	75.44	84.36	-0.11	0.65	-0.40	0.08	-0.56	0.00	-0.45	0.06	-0.29	0.30	0.16	0.47
ENSGALG00000011529	NAAA	56.57	74.50	49.14	60.29	-0.40	0.03	0.20	0.09	-0.09	0.53	0.30	0.13	0.60	0.00	0.29	0.04
ENSGALG00000012456	RAC2	56.40	77.18	47.41	45.85	-0.45	0.05	0.25	0.24	0.30	0.16	0.75	0.00	0.70	0.00	-0.05	0.76

Gene id	Gene name	CTL_FPKM	SP_FPKM	SPH_FPKM	SPM_FPKM	CTLvsSP_log$_2$FoldChange	CTLvsSP_Pvalue	CTLvsSPH_log$_2$FoldChange	CTLvsSPH_Pvalue	CTLvsSPM_log$_2$FoldChange	CTLvsSPM_Pvalue	SPvsSPM_log$_2$FoldChange	SPvsSPM_Pvalue	SPvsSPH_log$_2$FoldChange	SPvsSPH_Pvalue	SPMvsSPH_log$_2$FoldChange	SPMvsSPH_Pvalue
ENSGALG00000015663	HSDL2	56.14	78.23	80.69	73.78	-0.48	0.01	-0.52	0.00	-0.39	0.01	0.08	0.71	-0.05	0.85	-0.13	0.53
ENSGALG00000002605	MRPL17	55.70	75.64	86.26	71.36	-0.44	0.00	-0.63	0.01	-0.35	0.05	0.08	0.69	-0.19	0.46	-0.28	0.31
ENSGALG00000036395	IGHVL	55.43	81.33	50.59	53.07	-0.56	0.05	0.13	0.61	0.07	0.83	0.61	0.02	0.68	0.00	0.07	0.76
ENSGALG00000011738	ARHGDIB	55.38	76.20	51.95	49.17	-0.46	0.06	0.09	0.67	0.17	0.43	0.63	0.00	0.55	0.00	-0.08	0.60
ENSGALG00000054783	NDRG1	53.76	42.28	50.44	68.45	0.34	0.08	0.09	0.70	-0.35	0.16	-0.70	0.00	-0.26	0.29	0.44	0.11
ENSGALG00000007636	PCK1	53.35	57.19	53.92	95.70	-0.10	0.74	-0.02	0.95	-0.84	0.00	-0.74	0.04	0.08	0.79	0.83	0.01
ENSGALG00000005086	PLAU	53.04	48.77	43.06	67.58	0.12	0.51	0.30	0.07	-0.35	0.12	-0.47	0.04	0.18	0.30	0.65	0.00

Gene id	Gene name	CTL_FPKM	SP_FPKM	SPH_FPKM	SPM_FPKM	CTLvsSP_log2FoldChange	CTLvsSP_Pvalue	CTLvsSPH_log2FoldChange	CTLvsSPH_Pvalue	CTLvsSPM_log2FoldChange	CTLvsSPM_Pvalue	SPvsSPM_log2FoldChange	SPvsSPM_Pvalue	SPvsSPH_log2FoldChange	SPvsSPH_Pvalue	SPMvsSPH_log2FoldChange	SPMvsSPH_Pvalue
ENSGALG00000001475	STMN1	51.17	57.76	45.09	40.43	-0.18	0.31	0.18	0.27	0.34	0.05	0.51	0.00	0.36	0.02	-0.16	0.29
ENSGALG00000028998	XPA	50.95	73.95	78.02	67.40	-0.54	0.07	-0.62	0.00	-0.40	0.09	0.13	0.71	-0.08	0.85	-0.21	0.47
ENSGALG00000033683	PHOSPHO1	48.78	41.15	46.98	63.13	0.24	0.19	0.05	0.75	-0.37	0.03	-0.62	0.00	-0.19	0.30	0.42	0.01
ENSGALG00000028026	NDUFS4	48.46	75.81	80.73	63.69	-0.65	0.00	-0.74	0.01	-0.39	0.06	0.25	0.35	-0.09	0.83	-0.35	0.40
ENSGALG00000049973	TMEM252	48.44	62.99	92.23	56.57	-0.38	0.41	-0.93	0.02	-0.22	0.61	0.15	0.75	-0.55	0.24	-0.71	0.12
ENSGALG00000033862	TXNL1	48.22	69.95	66.73	58.92	-0.54	0.01	-0.47	0.01	-0.29	0.06	0.25	0.24	0.07	0.77	-0.18	0.38
ENSGALG00000028982	CMPK2	47.55	75.90	43.40	63.62	-0.68	0.01	0.13	0.64	-0.42	0.05	0.25	0.33	0.81	0.01	0.55	0.05

Gene id	Gene name	CTL_FPKM	SP_FPKM	SPH_FPKM	SPM_FPKM	CTLvsSP_log2FoldChange	CTLvsSP_Pvalue	CTLvsSPH_log2FoldChange	CTLvsSPH_Pvalue	CTLvsSPM_log2FoldChange	CTLvsSPM_Pvalue	SPvsSPM_log2FoldChange	SPvsSPM_Pvalue	SPvsSPH_log2FoldChange	SPvsSPH_Pvalue	SPMvsSPH_log2FoldChange	SPMvsSPH_Pvalue
ENSGALG00000016483	LOC421965	47.08	51.57	69.52	64.46	-0.13	0.56	-0.56	0.00	-0.45	0.01	-0.32	0.13	-0.43	0.04	-0.11	0.47
ENSGALG00000033857	NAT8B	46.97	39.75	49.10	62.48	0.24	0.15	-0.07	0.65	-0.41	0.01	-0.65	0.00	-0.31	0.01	0.35	0.00
ENSGALG00000028256	CCL19	44.37	75.36	54.53	41.84	-0.77	0.0007	-0.3	0.31	0.09	0.68	0.85	0.00	0.47	0.09	-0.38	0.14
